# Primary Lymphoma Presenting in an Ascending Aortic Aneurysm: A Case Report

**DOI:** 10.1055/s-0040-1701529

**Published:** 2020-07-31

**Authors:** Alexander M. Schurman, David Mendoza, Chris K. Rokkas

**Affiliations:** 1 Division of Cardiothoracic Surgery, Department of Surgery, Medical College of Wisconsin, Milwaukee, Wisconsin; 2 Department of Pathology, Medical College of Wisconsin, Milwaukee, Wisconsin

**Keywords:** aneurysm, aorta, lymphoma

## Abstract

Small lymphocytic lymphoma (SLL) is rarely associated with thoracic aortic aneurysms. Aneurysm of the ascending aorta associated with SLL has never been reported before. We describe the case of an asymptomatic 68-year-old woman who presented with a 5.5-cm aneurysm of the ascending aorta and no prior history of hematological disorders. Following excision and repair, the surgical specimen showed infiltration of the aortic wall by lymphocytes, expressing markers consistent with SLL. While symptomatic SLL carries a poor prognosis, risk stratification tools are applied to guide management strategies in asymptomatic patients.

## Introduction

Lymphoma associated with an aneurysm of the thoracic aorta is a rare occurrence. Few cases have been described and locations of the aneurysm are primarily confined to the descending thoracic aorta 1 2 3 4 5 or the aortic arch. 6 Small lymphocytic lymphoma (SLL) is a malignancy of B-lymphocytes that may present with constitutional symptoms and adenopathy. It is more prevalent in older adults and the prognosis is generally poor when symptomatic. In this report, we present the case of a patient who underwent surgical repair of an enlarging aneurysm confined to the ascending aorta and the proximal aortic arch. The surgical specimen contained histologic evidence of an infiltrative SLL. The patient had no prior history of SLL or other hematological disorders.

## Case Presentation

**Fig. 1:**
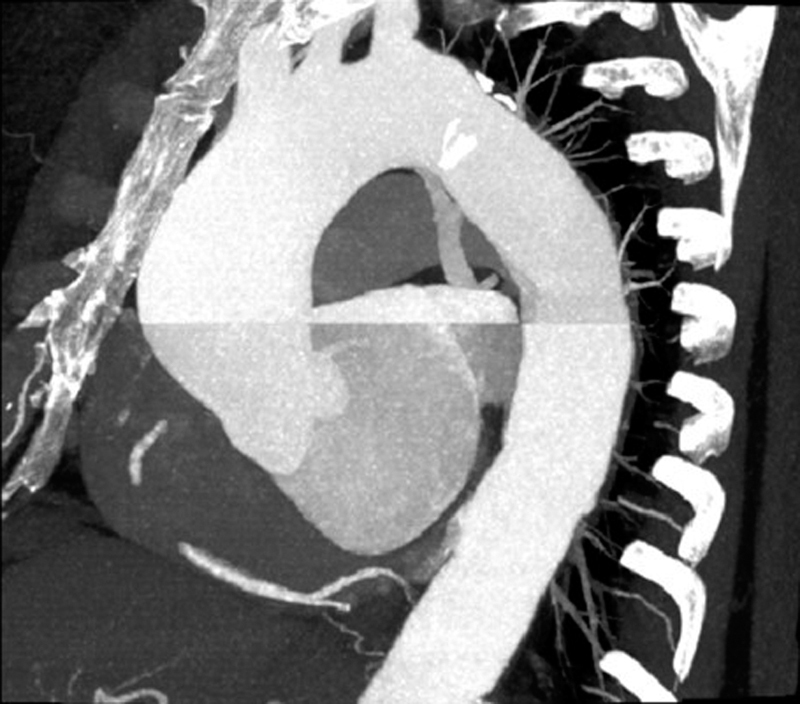
Computerized tomography angiogram.

A 68-year-old woman had a chest X-ray (CXR) that showed cardiomegaly following a fall at home. A subsequent echocardiogram revealed an enlarged ascending aorta with a greatest diameter of 5.0 cm. Surveillance computerized tomography angiogram (CTA), 6 months later, suggested enlargement of the aneurysm to 5.5 cm, prompting surgical consultation ( Fig. 1 ). The descending thoracic aorta was not enlarged. The aneurysmal ascending aorta was adjacent to the posterior plate of the sternum. A coronary angiogram was unremarkable. Routine hematological workup preoperatively was unremarkable.

Resection and replacement of the ascending aorta and the proximal aortic arch to just proximal to the origin of the innominate artery was performed uneventfully. There was no intraoperative evidence or suspicion of infiltration of the aortic wall by tumor. Femoral arterial and venous cannulation was implemented to facilitate a deep hypothermia and circulatory arrest perfusion strategy.

**Fig. 2:**
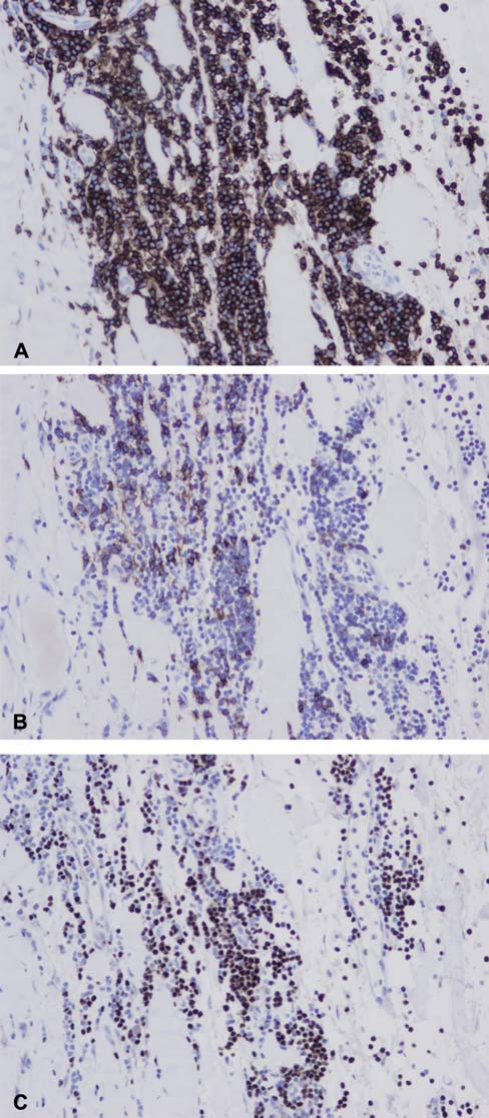
Immunohistological stains of adventitial infiltrate: ( **A** ) CD5, ( **B** ) CD20, and ( **C** ) PAX5.

**Fig. 3:**
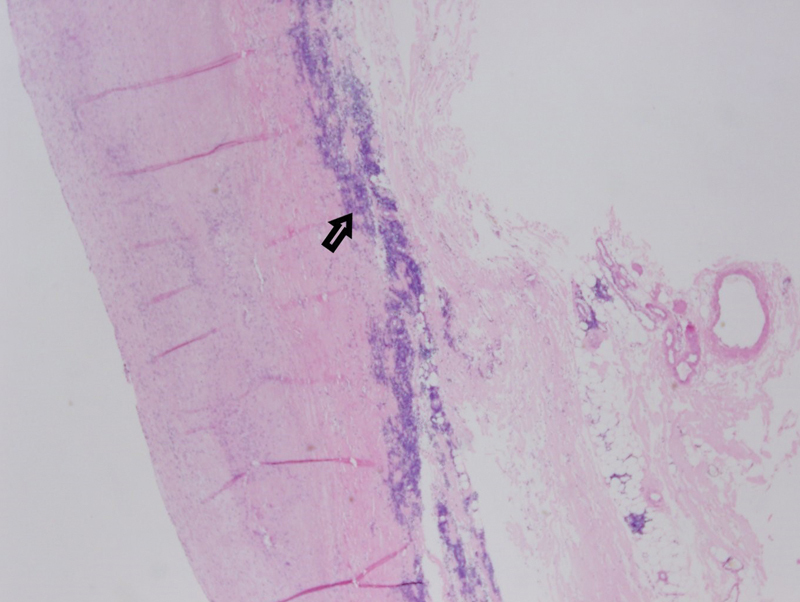
Low-power H&E stain showing the dense lymphocytic infiltration of the adventitia of the resected aortic segment (arrow).

Routine histopathologic examination of the resected ascending aorta showed dense lymphoid infiltrate on the adventitial aspect of the segment. The ectopic cells tested positive for CD5, PAX5, and CD20 consistent with SLL ( Fig. 2A–C ). Interestingly, the media of the aorta had some infiltration of both B- and T-lymphocytes. However, no significant evidence of medial degeneration was observed ( Fig. 3 ). Margins of aortic resection showed involvement by the same infiltrative process.

Our patient had an uncomplicated postoperative course. With respect to SLL, she was asymptomatic with no significant cytopenia prompting the oncology team to adopt a surveillance strategy for her management. At 14-month follow-up, she remained asymptomatic and she had no laboratory evidence of SLL. She will continue to follow-up regularly with cardiothoracic surgery and hematology–oncology.

## Discussion

Ascending aortic aneurysms are a result of degeneration of the medial layer of the aortic wall. Characteristically, this weakening occurs either through loss of smooth muscle cells or elastin fibers.

Thoracic aortic aneurysms associated with SLL have been previously described in the descending thoracic aorta. 1 2 3 4 5 To our knowledge, the association of a lymphoma with an ascending aortic aneurysm is novel. The extension of the aneurysm into the proximal aortic arch adds to the complexity of this case.

Our patient did not have a history of a heritable disease known to result in medial degeneration of the aorta, nor did she have a bicuspid aortic valve. Mycotic aneurysms are not an uncommon cause of ascending aortic aneurysm in the elderly. However, there was no evidence of infection in our surgical specimens. She had no prior history of hematological disorder. Histologically, the lymphocytic infiltrate was dense within the adventitia of the vessel. However, both CD5 and CD20 positive lymphocytes were noted within the media of the ascending aorta without associated medial degeneration. Etiologies for this patient's aneurysm may include: mycotic, reactive lymphocytosis, SLL, hypertension, and atherosclerosis. Her aneurysmal aorta was managed according to current size criteria. 7

The treatment for primary lymphomas of the vasculature is not well defined. Classically, treatment for small cell lymphoma includes chemotherapy appropriate for the signature of the malignant cells ( Fig. 2 ). Regimens may include a combination of purine analogs, such as fludarabine, and monoclonal antibodies (MOAB), such as the CD20-specific MOAB rituximab. The chemotherapy regimens for older patients, over the age of 70 years, may include a tyrosine kinase inhibitor, such as ibrutinib. The medial overall survival for patients with an isolated lymphoma is estimated between 3 and 8 years. Our multidisciplinary team chose a strategy of surveillance in this patient with an incidentally found lymphoma. This strategy will include hematological, as well as imaging testing.

The pathophysiology of ascending aorta aneurysms is known to be a result of medial degeneration. The presence of lymphoma on the adventitia, as well as B- and T-lymphocytes, within the media of the vessel may suggest either a resolving mycotic source for the aneurysm or a secondary process arising from reactive lymphocytosis. While symptomatic SLL carries a poor prognosis, most patients present without symptoms. Asymptomatic individuals may be observed for many years before chemotherapeutic management becomes necessary. Risk stratification tools, such as the Rai or Binet staging systems, can be utilized to guide initiation and susceptibilities of chemotherapeutic treatment. Chemotherapeutic management can consist of antimetabolites, such as fludarabine, or anti-CD20 antibodies, such as rituximab. 8

In conclusion, we presented the case of a woman with an incidentally discovered aneurysm of the ascending aorta that unexpectedly showed microscopic evidence of lymphocytic infiltration consistent with SLL. The role of this entity in the pathogenesis of aortic aneurysms remains unclear.
